# Supplementary material for: Genome-wide DNA methylation patterns in bumble bee (Bombus vosnesenskii) populations from spatial-environmental range extremes
Source: Sci Rep. 2023 Sep 9;13:14901. doi: 10.1038/s41598-023-41896-7 (PMC10492822; doi:10.1038/s41598-023-41896-7)
Supplement: Supplementary file 1 — Supplementary Information. [file 41598_2023_41896_MOESM1_ESM.docx]

**Supplemental Information for “Genome-wide DNA methylation patterns in bumble bee (*Bombus vosnesenskii*) populations from spatial-environmental range extremes*”***

**Supplementary Figures**

**Supplementary Figure 1:** Bar plots depicting exon and intron count and proportions for all CpG sites, unmethylated (<10%) sites, sparsely methylated (10-50%) sites, highly methylated sites (>50%) and differentially methylated sites (assessed at minimum 10% difference) present on long non-coding RNAs (lnc-RNAs).


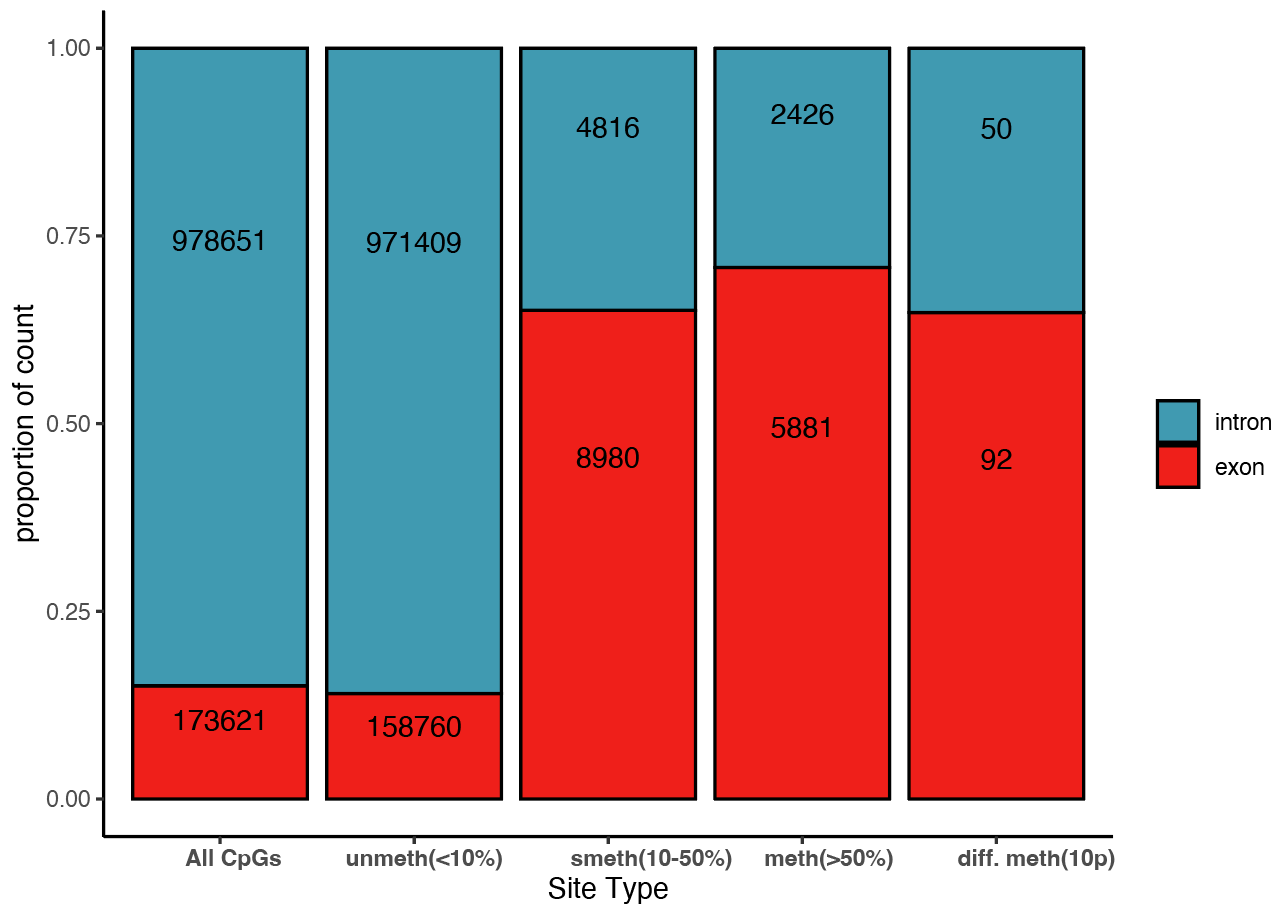


**Supplementary Figure 2:** Principal Component Analysis (PCA), scree plot representation of principal components, and hierarchical clustering plots for all CpG sites (row A), variable CpG sites (row B) and differentially methylated CpG sites (row C) in *B. vosnesenskii*.


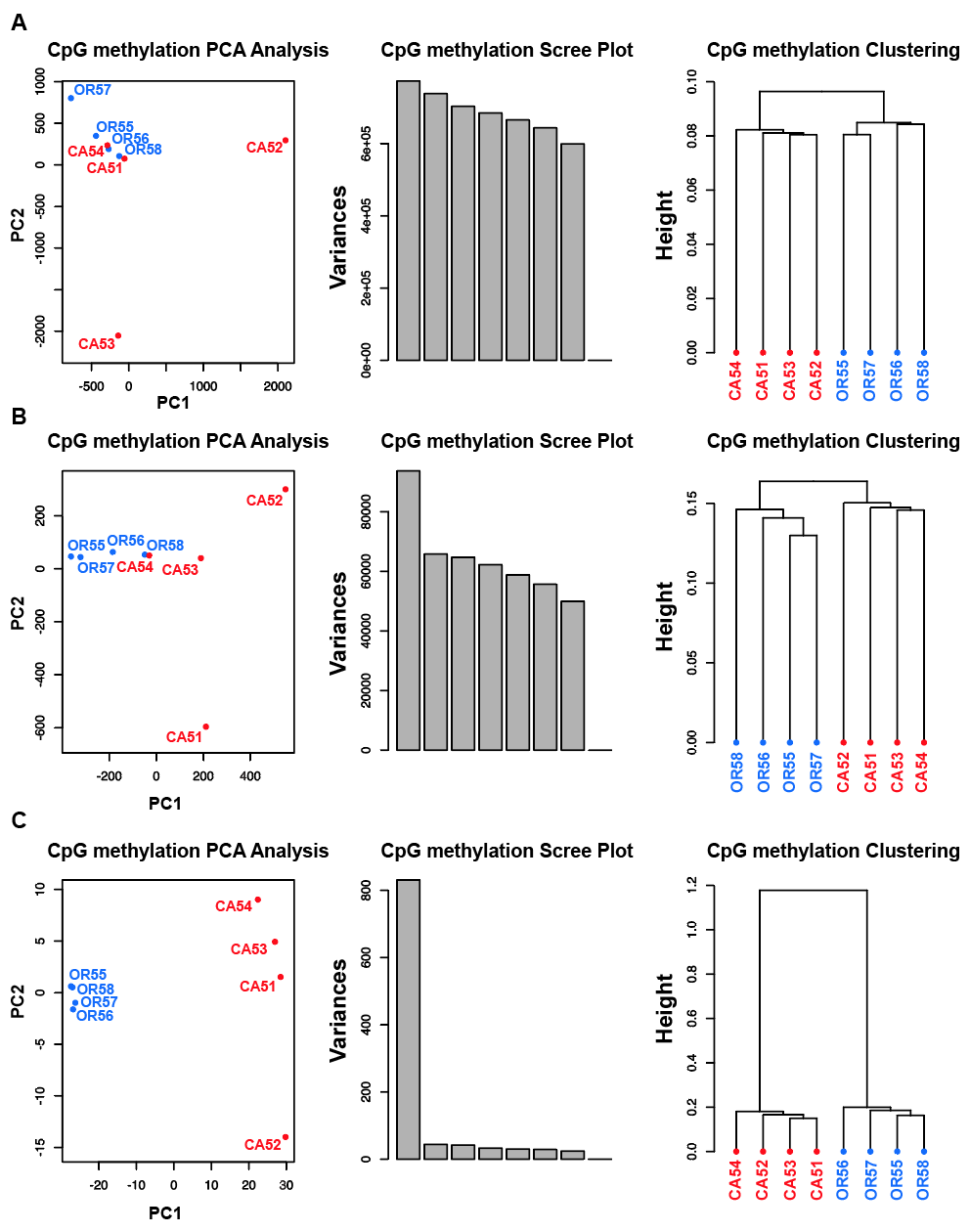


**Supplementary Tables**

**Supplementary Table 1:** Summary of results from a beta-binomial zero-inflated GLM (*glmmTMB* package in R) examining relationship between nucleotide diversity (π) and the proportion of highly methylated (>50% threshold) CpGs (out of all sequenced CpGs). See Figure 5A-B of main text. Simple correlations illustrating the slightly negative relationship are also presented in main Results.

|  | **cbind[Highly Methylated CpGs,** | | | |
| --- | --- | --- | --- | --- |
|  | **(total CpGs - Highly Methylated CpGs)]** | | | |
| *Predictors* | *Estimate* | *SE* | *Z* | *p* |
| **Count Model** |  |  |  |  |
| (Intercept) | -1.49 | 0.06 | -27.060 | **<0.001** |
| log(π) | -0.04 | 0.007 | -5.521 | **<0.001** |
| (Intercept) |  |  |  |  |
| **Zero-Inflated Model** |  |  |  |  |
| (Intercept) | 6.16 | 0.04 | 171.7 | **<0.001** |
| log(π) | 0.51 | 0.005 | 113.0 | **<0.001** |

N = 232,788

Dispersion parameter for betabinomial family = 4.84

Family: betabinomial(logit)

Formula: cbind(Highly Methylated CpG, total CpGs - Highly Methylated CpGs) ~ log(π)

Zero inflation: ~log(π)

R: glmm_tp_methcount_betabin<-glmmTMB(cbind(gen_meth_50, totalCpG-gen_meth_50)~logthetas, ziformula = ~logthetas, family=betabinomial, data=thetas2)

**Supplementary Table 2:** Statistically significant GO terms (n=18) from analysis of unique genes (n=44) harboring a minimum of 100 highly methylated sites.

| Ontology | Rank | GO ID | GO Term Description |
| --- | --- | --- | --- |
| Biological Process | 1 | GO:0000381 | regulation of alternative mRNA splicing, via spliceosome |
|  | 2 | GO:0048522 | positive regulation of cellular process |
|  | 3 | GO:0048523 | negative regulation of cellular process |
|  | 4 | GO:0048468 | cell development |
|  | 5 | GO:2000112 | regulation of cellular macromolecule biosynthetic process |
|  | 6 | GO:0010468 | regulation of gene expression |
|  | 7 | GO:1901315 | negative regulation of histone H2A K63-linked ubiquitination |
|  | 8 | GO:0030182 | neuron differentiation |
|  | 9 | GO:0051128 | regulation of cellular component organization |
|  | 10 | GO:0009892 | negative regulation of metabolic process |
|  | 11 | GO:0019219 | regulation of nucleobase-containing compound metabolic process |
| Molecular Function | 1 | GO:0003729 | mRNA binding |
|  | 2 | GO:0003723 | RNA binding |
| Cellular Component | 1 | GO:0016607 | nuclear speck |
|  | 2 | GO:1990904 | ribonucleoprotein complex |
|  | 3 | GO:0042382 | paraspeckles |
|  | 4 | GO:0035061 | interchromatin granule |
|  | 5 | GO:0005726 | perichromatin fibrils |

**Supplementary Table 3:** Summarized GO Clusters from analysis of unique genes (n=44) harboring a minimum of 100 highly methylated sites.

| Ontology | Rank | GO ID | GO Term Description |
| --- | --- | --- | --- |
| Biological Process | 1 | GO:0048522 | positive regulation of cellular process |
|  | 2 | GO:0048523 | negative regulation of cellular process |
|  | 3 | GO:0048468 | cell development |
| Molecular Function | 1 | GO:0003729 | mRNA binding |
| Cellular Component | 1 | GO:0016607 | nuclear speck |
|  | 2 | GO:1990904 | ribonucleoprotein complex |
|  | 3 | GO:0005726 | perichromatin fibrils |

**Supplementary Table 4:** A list of statistically significant GO terms from analysis (n=89) of all unique genes (n = 1,272) harboring differentially methylated sites (n = 2,066) assessed at minimum 10% methylation difference.

| Ontology | Rank | GO ID | GO Term Description |
| --- | --- | --- | --- |
| Biological Process | 1 | GO:1902531 | regulation of intracellular signal transduction |
|  | 2 | GO:0016458 | gene silencing |
|  | 3 | GO:0048513 | animal organ development |
|  | 4 | GO:0009968 | negative regulation of signal transduction |
|  | 5 | GO:0007292 | female gamete generation |
|  | 6 | GO:0010564 | regulation of cell cycle process |
|  | 7 | GO:0006403 | RNA localization |
|  | 8 | GO:0006468 | protein phosphorylation |
|  | 9 | GO:0040008 | regulation of growth |
|  | 10 | GO:0016573 | histone acetylation |
|  | 11 | GO:0010604 | positive regulation of macromolecule metabolic process |
|  | 12 | GO:0043933 | protein-containing complex subunit organization |
|  | 13 | GO:0032268 | regulation of cellular protein metabolic process |
|  | 14 | GO:0007346 | regulation of mitotic cell cycle |
|  | 15 | GO:0007028 | cytoplasm organization |
|  | 16 | GO:0006914 | autophagy |
|  | 17 | GO:0051128 | regulation of cellular component organization |
|  | 18 | GO:0051129 | negative regulation of cellular component organization |
|  | 19 | GO:0051225 | spindle assembly |
|  | 20 | GO:0051172 | negative regulation of nitrogen compound metabolic process |
|  | 21 | GO:0050807 | regulation of synapse organization |
|  | 22 | GO:0006886 | intracellular protein transport |
|  | 23 | GO:0030707 | ovarian follicle cell development |
|  | 24 | GO:0044087 | regulation of cellular component biogenesis |
|  | 25 | GO:0040029 | regulation of gene expression, epigenetic |
|  | 26 | GO:0071103 | DNA conformation change |
|  | 27 | GO:0022607 | cellular component assembly |
|  | 28 | GO:0009653 | anatomical structure morphogenesis |
|  | 29 | GO:0051656 | establishment of organelle localization |
|  | 30 | GO:0007349 | cellularization |
|  | 31 | GO:0006355 | regulation of transcription, DNA-templated |
|  | 32 | GO:0019941 | modification-dependent protein catabolic process |
|  | 33 | GO:0016358 | dendrite development |
|  | 34 | GO:0007308 | oocyte construction |
|  | 35 | GO:0043044 | ATP-dependent chromatin remodeling |
|  | 36 | GO:0007167 | enzyme linked receptor protein signaling pathway |
|  | 37 | GO:0051231 | spindle elongation |
|  | 38 | GO:0022411 | cellular component disassembly |
|  | 39 | GO:0031324 | negative regulation of cellular metabolic process |
|  | 40 | GO:0000380 | alternative mRNA splicing, via spliceosome |
|  | 41 | GO:0051094 | positive regulation of developmental process |
|  | 42 | GO:0043161 | proteasome-mediated ubiquitin-dependent protein catabolic process |
|  | 43 | GO:0043547 | positive regulation of GTPase activity |
|  | 44 | GO:0016477 | cell migration |
|  | 45 | GO:0048522 | positive regulation of cellular process |
|  | 46 | GO:0032954 | regulation of cytokinetic process |
|  | 47 | GO:0048312 | intracellular distribution of mitochondria |
|  | 48 | GO:0140014 | mitotic nuclear division |
|  | 49 | GO:0033554 | cellular response to stress |
|  | 50 | GO:0006997 | nucleus organization |
|  | 51 | GO:0000819 | sister chromatid segregation |
|  | 52 | GO:0007281 | germ cell development |
|  | 53 | GO:0051493 | regulation of cytoskeleton organization |
|  | 54 | GO:0048024 | regulation of mRNA splicing, via spliceosome |
|  | 55 | GO:0045880 | positive regulation of smoothened signaling pathway |
| Molecular Function | 1 | GO:0003682 | chromatin binding |
|  | 2 | GO:0005524 | ATP binding |
|  | 3 | GO:0003723 | RNA binding |
|  | 4 | GO:0004386 | helicase activity |
|  | 5 | GO:0004674 | protein serine/threonine kinase activity |
|  | 6 | GO:0019901 | protein kinase binding |
|  | 7 | GO:0005515 | protein binding |
|  | 8 | GO:0019899 | enzyme binding |
|  | 9 | GO:0046872 | metal ion binding |
|  | 10 | GO:0003729 | mRNA binding |
|  | 11 | GO:0005096 | GTPase activator activity |
|  | 12 | GO:0044877 | protein-containing complex binding |
|  | 13 | GO:0045296 | cadherin binding |
|  | 14 | GO:0003779 | actin binding |
|  | 15 | GO:0003677 | DNA binding |
|  | 16 | GO:0042393 | histone binding |
|  | 17 | GO:0003678 | DNA helicase activity |
| Cellular Component | 1 | GO:0005829 | cytosol |
|  | 2 | GO:0005654 | nucleoplasm |
|  | 3 | GO:0016607 | nuclear speck |
|  | 4 | GO:0140513 | nuclear protein-containing complex |
|  | 5 | GO:0005700 | polytene chromosome |
|  | 6 | GO:0016234 | inclusion body |
|  | 7 | GO:0031410 | cytoplasmic vesicle |
|  | 8 | GO:0072686 | mitotic spindle |
|  | 9 | GO:0048471 | perinuclear region of cytoplasm |
|  | 10 | GO:0005794 | Golgi apparatus |
|  | 11 | GO:0099738 | cell cortex region |
|  | 12 | GO:0005815 | microtubule organizing center |
|  | 13 | GO:0030496 | midbody |
|  | 14 | GO:0005703 | polytene chromosome puff |
|  | 15 | GO:0010494 | cytoplasmic stress granule |
|  | 16 | GO:0005730 | nucleolus |
|  | 17 | GO:0045169 | fusome |

**Supplementary Table 5:** Summarized GO Clusters from analysis of all unique genes (n = 1,272) harboring differentially methylated sites (n = 2,066) assessed at minimum 10% methylation difference.

| Ontology | Rank | Representative GO ID | GO Term Description |
| --- | --- | --- | --- |
| Biological Process | 1 | GO:0016458 | gene silencing |
|  | 2 | GO:0048513 | animal organ development |
|  | 3 | GO:0007292 | female gamete generation |
|  | 4 | GO:0006403 | RNA localization |
|  | 5 | GO:0006468 | protein phosphorylation |
|  | 6 | GO:0040008 | regulation of growth |
|  | 7 | GO:0006914 | autophagy |
|  | 8 | GO:0051225 | spindle assembly |
|  | 9 | GO:0000380 | alternative mRNA splicing, via spliceosome |
|  | 10 | GO:0016477 | cell migration |
|  | 11 | GO:0033554 | cellular response to stress |
| Molecular Function | 1 | GO:0003682 | chromatin binding |
|  | 2 | GO:0003723 | RNA binding |
|  | 3 | GO:0004386 | helicase activity |
|  | 4 | GO:0004674 | protein serine/threonine kinase activity |
|  | 5 | GO:0019901 | protein kinase binding |
|  | 6 | GO:0005096 | GTPase activator activity |
|  | 7 | GO:0044877 | protein-containing complex binding |
| Cellular Component | 1 | GO:0005829 | cytosol |
|  | 2 | GO:0005654 | nucleoplasm |
|  | 3 | GO:0016607 | nuclear speck |
|  | 4 | GO:0140513 | nuclear protein-containing complex |
|  | 5 | GO:0005700 | polytene chromosome |
|  | 6 | GO:0016234 | inclusion body |
|  | 7 | GO:0031410 | cytoplasmic vesicle |
|  | 8 | GO:0048471 | perinuclear region of cytoplasm |
|  | 9 | GO:0099738 | cell cortex region |
|  | 10 | GO:0005815 | microtubule organizing center |
|  | 11 | GO:0030496 | midbody |
|  | 12 | GO:0005703 | polytene chromosome puff |
|  | 13 | GO:0045169 | fusome |

**Supplementary Table 6:** A list of overlapping statistically significant GO terms (n=5) between highly methylated and differentially methylated CpG harboring Gene Sets.

| Ontology | GO Term ID | GO Term Description |
| --- | --- | --- |
| Biological Process | GO:0048522 | positive regulation of cellular process |
|  | GO:0051128 | regulation of cellular component organization |
| Molecular Function | GO:0003723 | RNA binding |
|  | GO:0003729 | mRNA binding |
| Cellular Component | GO:0016607 | nuclear speck |

*** There is one overlapping summarized GO cluster [nuclear speck (Representative GO ID|GO:0016607, Cellular component] between highly methylated and differentially methylated CpG harboring Gene Sets.

**Supplementary Table 7:** Comparison of the GO term lists from this study with two previous thermal tolerance studies [Pimsler et al. 2020 and Jackson et al. 2020; refs 56 and 55 on main text references] in bumble bees. For detailed procedure of the comparison, see the “Gene Ontology (GO) analysis of highly methylated and differentially methylated CpGs” subsection of the main text Methods.

| Comparison Type | Ontology | GO ID | GO Term Description |
| --- | --- | --- | --- |
| Overlapping GO terms from Highly methylated CpG harboring gene set with Pimsler et al. 2020 (n=10) | Biological Process | GO:0000381 | regulation of alternative mRNA splicing, via spliceosome |
|  |  | GO:0048522 | positive regulation of cellular process |
|  |  | GO:0048523 | negative regulation of cellular process |
|  |  | GO:0048468 | cell development |
|  |  | GO:2000112 | regulation of cellular macromolecule biosynthetic process |
|  |  | GO:0010468 | regulation of gene expression |
|  |  | GO:0030182 | neuron differentiation |
|  |  | GO:0009892 | negative regulation of metabolic process |
|  |  | GO:0019219 | regulation of nucleobase-containing compound metabolic process |
|  | Cellular Component | GO:0016607 | nuclear speck |
| Overlapping GO terms from Differentially methylated CpG harboring gene set with Pimsler et al. 2020 (n=34) | Biological Process | GO:0000380 | alternative mRNA splicing, via spliceosome |
|  |  | GO:0006355 | regulation of transcription, DNA-templated |
|  |  | GO:0006914 | autophagy |
|  |  | GO:0007167 | enzyme linked receptor protein signaling pathway |
|  |  | GO:0007281 | germ cell development |
|  |  | GO:0007292 | female gamete generation |
|  |  | GO:0007308 | oocyte construction |
|  |  | GO:0009653 | anatomical structure morphogenesis |
|  |  | GO:0016358 | dendrite development |
|  |  | GO:0016458 | gene silencing |
|  |  | GO:0016477 | cell migration |
|  |  | GO:0022607 | cellular component assembly |
|  |  | GO:0030707 | ovarian follicle cell development |
|  |  | GO:0040029 | regulation of gene expression, epigenetic |
|  |  | GO:0043161 | proteasome-mediated ubiquitin-dependent protein catabolic process |
|  |  | GO:0043933 | protein-containing complex subunit organization |
|  |  | GO:0044087 | regulation of cellular component biogenesis |
|  |  | GO:0048024 | regulation of mRNA splicing, via spliceosome |
|  |  | GO:0048513 | animal organ development |
|  |  | GO:0048522 | positive regulation of cellular process |
|  |  | GO:0050807 | regulation of synapse organization |
|  |  | GO:0051129 | negative regulation of cellular component organization |
|  |  | GO:0071103 | DNA conformation change |
|  |  | GO:1902531 | regulation of intracellular signal transduction |
|  | Molecular Function | GO:0005515 | protein binding |
|  |  | GO:0005524 | ATP binding |
|  |  | GO:0044877 | protein-containing complex binding |
|  | Cellular Component | GO:0005654 | nucleoplasm |
|  |  | GO:0005700 | polytene chromosome |
|  |  | GO:0005703 | polytene chromosome puff |
|  |  | GO:0010494 | cytoplasmic stress granule |
|  |  | GO:0016607 | nuclear speck |
|  |  | GO:0045169 | fusome |
|  |  | GO:0048471 | perinuclear region of cytoplasm |
| Overlapping GO terms from Highly methylated CpG harboring gene set with Jackson et al. 2020 (n= 0) | NA | NA | NA |
| Overlapping GO terms from Differentially methylated CpG harboring gene set with Jackson et al. 2020 (n= 5) | Biological Process | GO:0009968 | negative regulation of signal transduction |
|  |  | GO:0016458 | gene silencing |
|  |  | GO:0040029 | regulation of gene expression, epigenetic |
|  | Molecular Function | GO:0003682 | chromatin binding |
|  |  | GO:0046872 | metal ion binding |

| **Sample Name and Locality**  **(OR= Oregon; CA= California)** | **NCBI SRA RUN ID** | **No. of Raw read pairs (in millions)** | **Raw reads pairs (in Gbp)** | **No. of Trimmed read pairs (in millions)** | **Trimmed read pairs (in Gbp)** | **No. of Read pairs after subsampling** | **Read pairs after subsampling (in Gbp)** | **Sequencing Depth after Read Mapping** | **% deduplicated reads** | **No. of Bioinformatic removed likely variants** | **Methyldackel output CpGs (in millions)** | **post-filtering CpGs (after removing <10X & >99th perc. Cov.) (in millions)** | **United CpG set present in all samples** | **No. of Genomic SNPs overlapping CpGs** | **No. of CpGs after Genomic SNP removal** | **No. of variable CpG sites (sd >2)** | **% average genome wide CpG Methylation** |
| --- | --- | --- | --- | --- | --- | --- | --- | --- | --- | --- | --- | --- | --- | --- | --- | --- | --- |
| Bvo_JDL3147-OR052016_SL303655 | SRR24182864 | 476.76 | 143.98 | 428.83 | 76.24 | 187,618,210 read pairs  (For each sample) | 33.35 | 74.60 | 0.16 | 58,664 | 20.04 | 19.84 | 14,627,533 | 44,041 | 14,583,492 | 901,868 | 0.99 |
| Bvo_JDL3148-OR052016_SL303656 | SRR24182863 | 436.34 | 131.77 | 378.78 | 66.96 |  | 33.17 | 72.61 | 0.21 | 67,323 | 23.35 | 23.11 |  |  |  |  | 1.04 |
| Bvo_JDL3150-OR052016_SL303657 | SRR24182862 | 495.92 | 149.77 | 446.13 | 79.37 |  | 33.38 | 75.82 | 0.14 | 64,127 | 22.17 | 21.94 |  |  |  |  | 1.00 |
| Bvo_JDL3152-OR052016_SL303658 | SRR24182861 | 335.46 | 101.31 | 296.43 | 52.55 |  | 33.26 | 70.70 | 0.22 | 56,815 | 19.12 | 18.92 |  |  |  |  | 1.08 |
| Bvo_JDL928-CA012015_SL303652 | SRR24182860 | 480.41 | 145.08 | 214.13 | 37.69 |  | 33.02 | 78.50 | 0.17 | 68,939 | 23.94 | 23.69 |  |  |  |  | 1.24 |
| Bvo_JDL929-CA012015_SL303653 | SRR24182859 | 470.17 | 141.99 | 209.72 | 36.87 |  | 32.99 | 78.25 | 0.15 | 70,584 | 24.23 | 23.98 |  |  |  |  | 1.17 |
| Bvo_JDL931-CA012015_SL303654 | SRR24182858 | 462.25 | 139.60 | 205.54 | 35.99 |  | 32.86 | 79.54 | 0.21 | 69,503 | 23.59 | 23.35 |  |  |  |  | 1.11 |
| Bvo_JDL940-CA012015_SL303651 | SRR24182857 | 444.22 | 134.16 | 187.62 | 32.94 |  | 32.94 | 75.60 | 0.21 | 62,826 | 21.08 | 20.87 |  |  |  |  | 1.15 |
| Total |  | 3,601.52m | 1087.66 | 2,367.19 | 418.60 | 1,500.95m | 264.96 | 605.63 | 1.47 | 518,781 | 177.51 | 175.69 |  |  |  |  | 8.78 |
| Mean |  | 450.19 | 135.96 | 295.90 | 52.33 | 187.62m | 33.12 | 75.70 | 0.18 | 64,847.63 | 22.19 | 21.96 |  |  |  |  | 1.10 |
| Standard Deviation |  | 50.21 | 15.16 | 107.65 | 19.32 | 0.00m | 0.20 | 3.04 | 0.03 | 5,138.26 | 1.92 | 1.90 |  |  |  |  | 0.09 |

**Supplementary Table 8:** Summary Statistics from CPG methylation data analysis pipeline. See “Methods” section for detailed analysis procedures.
